# The role of interleukin 17 in Crohn’s disease-associated intestinal fibrosis

**DOI:** 10.1186/1755-1536-6-13

**Published:** 2013-07-08

**Authors:** Paolo Biancheri, Sylvia LF Pender, Francesca Ammoscato, Paolo Giuffrida, Gianluca Sampietro, Sandro Ardizzone, Amir Ghanbari, Renata Curciarello, Alessandra Pasini, Giovanni Monteleone, Gino R Corazza, Thomas T MacDonald, Antonio Di Sabatino

**Affiliations:** 1 Centre for Immunology and Infectious Disease, Blizard Institute, Barts and the London School of Medicine and Dentistry, London, UK; 2 Department of Internal Medicine, S. Matteo Hospital, Centro per lo Studio e la Cura delle Malattie Infiammatorie Croniche Intestinali, University of Pavia, Pavia, Italy; 3 Division of Infection, Inflammation and Immunity, University of Southampton, Southampton, UK; 4 Surgery Division, Department of Clinical Sciences, L. Sacco University Hospital, Milan, Italy; 5 Gastrointestinal Unit, Department of Clinical Sciences, L. Sacco University Hospital, Milan, Italy; 6 Department of Systems Medicine, University of Rome Tor Vergata, Rome, Italy; 7 Blizard Institute, Barts and The London School of Medicine and Dentistry, E1 2AT London, UK

**Keywords:** Collagen, Interleukin 25, Myofibroblast, Stricture, TIMP-1

## Abstract

**Background:**

Interleukin (IL)-17A and IL-17E (also known as IL-25) have been implicated in fibrosis in various tissues. However, the role of these cytokines in the development of intestinal strictures in Crohn’s disease (CD) has not been explored. We investigated the levels of IL-17A and IL-17E and their receptors in CD strictured and non-strictured gut, and the effects of IL-17A and IL-17E on CD myofibroblasts.

**Results:**

IL-17A was significantly overexpressed in strictured compared with non-strictured CD tissues, whereas no significant difference was found in the expression of IL-17E or IL-17A and IL-17E receptors (IL-17RC and IL-17RB, respectively) in strictured and non-strictured CD areas. Strictured CD explants released significantly higher amounts of IL-17A than non-strictured explants, whereas no difference was found as for IL-17E, IL-6, or tumor necrosis factor-α production. IL-17A, but not IL-17E, significantly inhibited myofibroblast migration, and also significantly upregulated matrix metalloproteinase (MMP)-3, MMP-12, tissue inhibitor of metalloproteinase-1 and collagen production by myofibroblasts from strictured CD tissues.

**Conclusions:**

Our results suggest that IL-17A, but not IL-17E, is pro-fibrotic in CD. Further studies are needed to clarify whether the therapeutic blockade of IL-17A through the anti-IL-17A monoclonal antibody secukinumab is able to counteract the fibrogenic process in CD.

## Background

Fibrosis is the common end-stage of chronic inflammatory diseases in many tissues. There is excessive accumulation of extracellular matrix (ECM) components, including collagen and fibronectin, in the damaged tissue, leading to scarring and organ malfunction [1]. ECM-producing myofibroblasts are the key players of fibrotic tissue remodeling,and consequently, much of the fibrosis research has focused on elucidating the molecular and immunological mechanisms underlying myofibroblast activation, proliferation, and migration [2].

A number of cytokines can modulate myofibroblast function, including those belonging to the interleukin (IL)-17 family [3,4]. In particular, IL-17A stimulates proliferation and migration of cardiac fibroblasts [5], promotes hepatic stellate cell activation into fibrogenic myofibroblasts [6], induces collagen production by skin fibroblasts [7], and promotes epithelial-mesenchymal transition of lung epithelial cells [8]. IL-17E, also known as IL-25, upregulates pro-inflammatory cytokine expression and collagen production by lung fibroblasts [9,10].

We recently showed that IL-17A is increased in the inflamed areas of patients with inflammatory bowel disease (IBD) [11]. By contrast, expression of IL-17E, which exerts an anti-inflammatory action by inhibiting T helper (Th)1 and Th17 responses, is markedly reduced in the inflamed mucosa of patients with IBD [12]. However, there is much less clarity about the influence that these cytokines exert on intestinal fibrosis, which is a frequent complication in Crohn’s disease (CD) [13].

Intestinal myofibroblasts are central to CD fibrosis [14,15]. Myofibroblasts isolated from strictured gut tissue of patients with CD overexpress collagen and transforming growth factor (TGF)-β1, produce excessive amounts of tissue inhibitor of metalloproteinases (TIMP)-1, and display reduced migratory ability compared with myofibroblasts isolated from non-strictured intestinal areas in CD [16]. Although intestinal myofibroblasts are the target of a number of pro-inflammatory cytokines, including IL-1β and tumor necrosis factor (TNF)-α [17], no information is available on expression of the IL-17A and IL-17E receptors (IL-17RC and IL-17RB respectively), or on the effect of IL-17A and IL-17E on the release of pro-fibrogenic mediators by these cells.

Thus, in this study, we assessed expression of IL-17A and IL-17E in CD strictured and non-strictured gut, and investigated IL-17A and IL-17E production by CD strictured tissue explants cultured *ex vivo*. In addition, we assessed IL-17A and IL-17E receptor expression in CD strictured and non-strictured tissue and on myofibroblasts from the same areas, and performed *in vitro* experiments to determine the effects of IL-17A and IL-17E on CD myofibroblasts.

## Results

### *In vivo* IL-17A and IL-17E tissue expression

**Figure 1:**
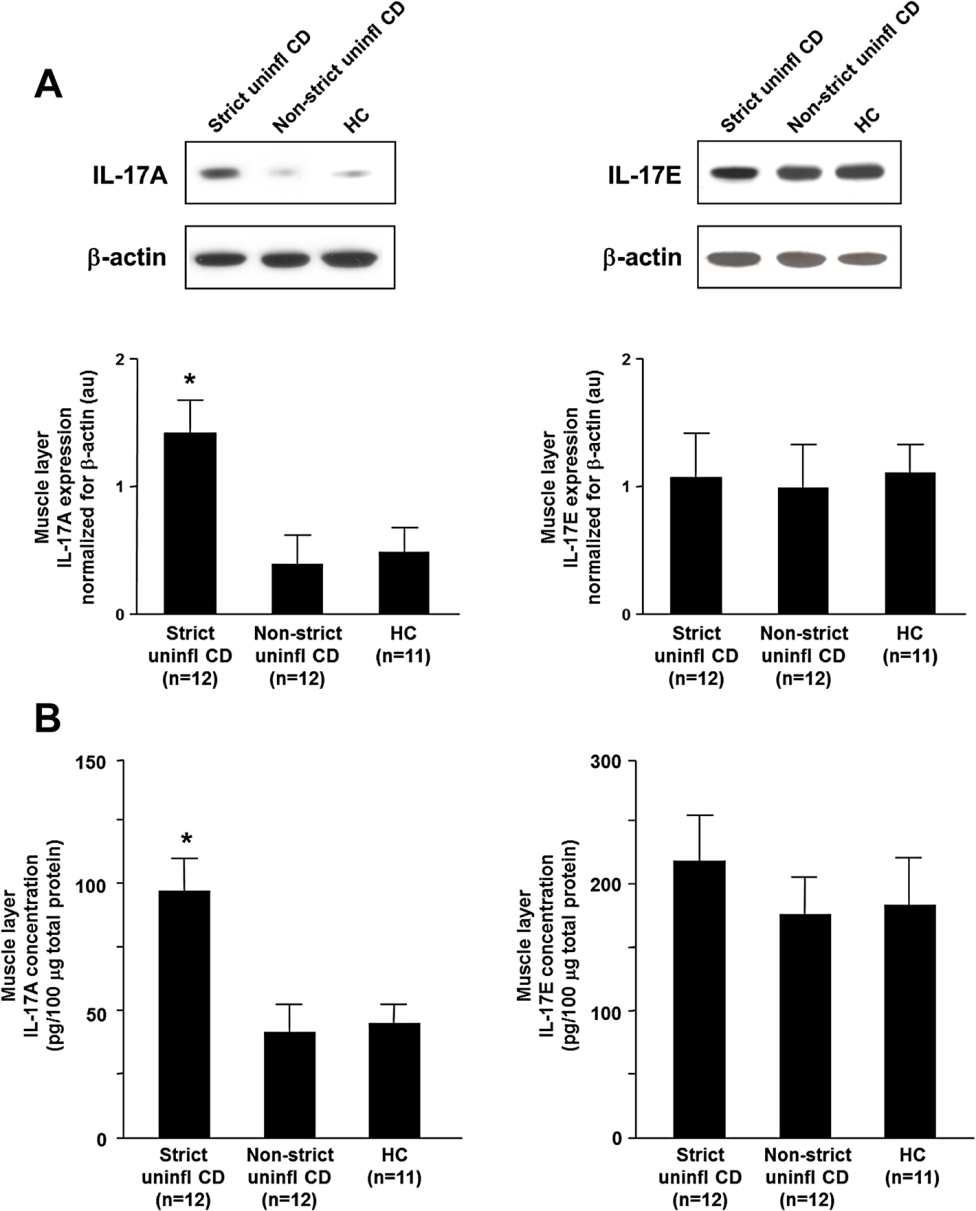
***In vivo *expression of interleukin (IL)-17A and IL-17E.** IL-17A and IL-17E were detected by both **(A)** immunoblotting and **(B)** ELISA in uninflamed areas of strictured (Strict uninfl) and non-strictured (Non-strict uninfl) gut of 12 patients with fibrostenosing Crohn’s disease (CD) and from normal gut of 11 healthy control (HC) subjects. **(A)** Each example shown in the upper panel is representative of experiments performed in 12 patients with CD and 11 HC subjects. Blots were stripped and analyzed for β-actin as an internal loading control. In the lower panel, densitometry of IL-17A and IL-17E expression normalized for β-actin is shown. Results are mean ± SEM. au, Arbitrary units. **(B)** Results, expressed as pg/100 μg of total protein, are mean ± SEM. *P<0.005 versus Non-strict uninfl and HC tissue samples.

We analyzed IL-17A and IL-17E levels by immunoblotting in tissue samples collected from uninflamed areas of strictured and non-strictured gut of 12 patients with fibrostenosing CD and from normal gut of 11 control subjects. IL-17A expression was significantly (*P*<0.005) upregulated in strictured CD areas compared with non-strictured CD areas and control gut (Figure 1A). No significant difference in IL-17A expression was found between non-strictured CD areas and control gut. IL-17E levels did not differ significantly between strictured and non-strictured CD areas and control gut.

In parallel, we analyzed IL-17A and IL-17E expression by ELISA in the same samples. IL-17A was significantly upregulated in strictured CD (mean 98.5 ± 13.7 pg/μg total protein) compared with non-strictured CD (mean 42.3 ± 11.0 pg/μg total protein, *P*<0.005) and control (mean 46.5 ± 8.3 pg/μg total protein, *P*<0.005) areas (Figure 1B). There was no significant difference in IL-17A expression between non-strictured CD areas and control gut. IL-17E did not significantly differ between strictured CD (mean 222.5 ± 32.4 pg/μg total protein), non-strictured CD (mean 178.1 ± 30.3 pg/μg total protein), and control areas (mean 188.2 ± 35.3 pg/μg total protein).

### *In vivo* IL-17RC and IL-17RB tissue expression

**Figure 2:**
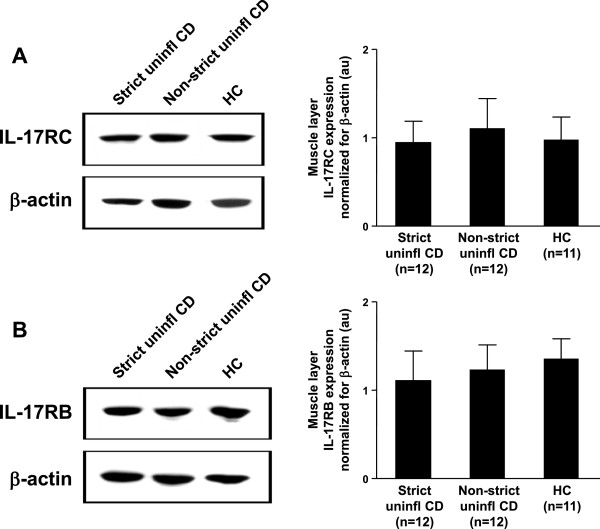
***In vivo *expression of interleukin (IL)-17A and IL-17E receptors. (A)** IL-17RC (receptor of IL-17A), and **(B)** IL-17RB (receptor of IL-17E) were detected by immunoblotting in uninflamed areas of strictured (Strict uninfl) and non-strictured (Non-strict uninfl) gut of 12 patients with fibrostenosing Crohn’s disease (CD) and from normal gut of 11 healthy control (HC)subjects. Each example shown in the left panels is representative of experiments performed in all patients with CD and all HC subjects. Blots were stripped and analyzed for β-actin as an internal loading control. In the right panels, densitometry of IL-17RC and IL-17RB expression normalized for β-actin is shown. Results are mean ± SEM. au, Arbitrary units.

We then analyzed IL-17RC (receptor of IL-17A) and IL-17RB (receptor of IL-17E) expression by immunoblotting in the same tissue samples. IL-17RC expression was not significantly different between CD strictured, CD non-strictured, and control gut (Figure 2A). Similarly, IL-17RB expression did not differ between CD strictured, CD non-strictured and control gut (Figure 2B).

### *Ex vivo* tissue production of IL-17A, IL-17E, IL-6, TNF-α, collagen and TGF-β1

**Figure 3:**
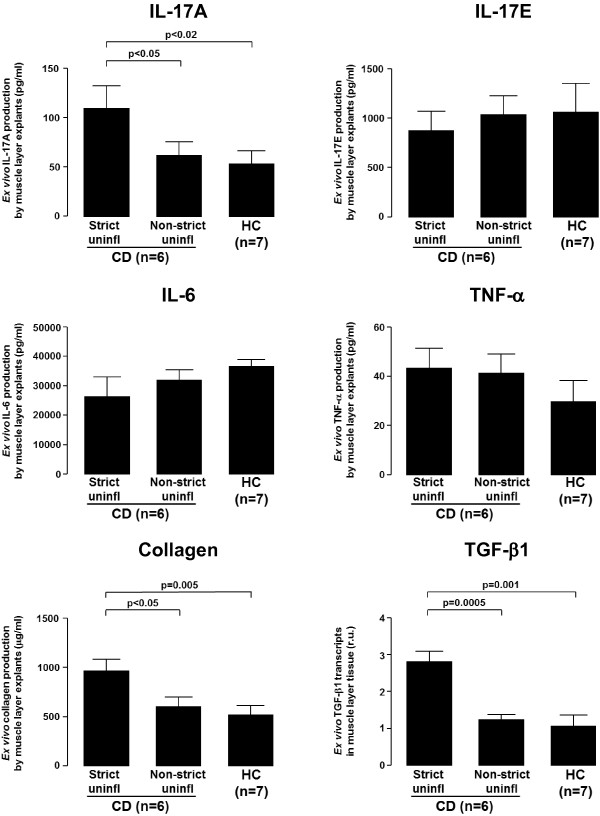
**Levels of cytokines and pro-fibrogenic mediators in tissue explant organ culture supernatants.** Levels of interleukin (IL)-17A, IL-17E, IL-6, and tumor necrosis factor (TNF)-α, expressed as pg/ml, and collagen, expressed as μg/ml, in the supernatants of tissue explants from uninflamed areas of strictured (Strict uninfl) and non-strictured (Non-strict uninfl) gut of six patients with fibrostenosing Crohn’s disease (CD) and from normal gut of seven control subjects (HC), cultured for 24 hours in the absence of stimuli, and levels of transforming growth factor (TGF)-β1, expressed as relative units compared with the median expression in control subjects (which was assigned the value 1), in the same cultured tissue explants. Values are mean ± SEM. r.u., Relative units.

We cultured *ex vivo* for 24 hours tissue explants (1 mm^3^ in size) from uninflamed areas of strictured and non-strictured gut of six patients with fibrostenosing CD and from normal gut of seven control subjects, and measured IL-17A, IL-17E, IL-6, TNF-α, and collagen concentration in the culture supernatant and TGF-β1 transcripts in the cultured tissue (Figure 3). IL-17A was significantly higher in the organ culture supernatants of strictured CD (mean 110.0 ± 23.8 pg/ml) than in those of non-strictured CD (mean 55.9 ± 8.8 pg/ml, *P*<0.05) or normal gut (mean 51.1 ± 8.9 pg/ml, *P*<0.02). Concentrations of IL-17E, IL-6, and TNF-α did not differ significantly between the supernatants of strictured CD (mean 653.3 ± 283.5 pg/ml, 26,553 ± 6647 pg/ml, and 43.4 ± 7.6 pg/ml, respectively), non-strictured CD (mean 1,028.0 ± 202.5 pg/ml, 31,740 ± 3944 pg/ml, and 40.8 ± 7.7 pg/ml, respectively), and normal gut (mean 1068.0 ± 282.8 pg/ml, 36,784 ± 2516 pg/ml, and 30.0 ± 7.9 pg/ml, respectively). Collagen concentration was significantly higher in the supernatants of strictured CD (mean 979.0 ± 108.6 μg/ml, respectively) than in those of non-strictured CD (mean 604.6 ± 97.5 μg/ml, *P*<0.05) and normal gut areas (mean 523.6 ± 77.9 μg/ml, *P*=0.005). TGF-β1 transcripts were significantly higher in the cultured tissue explants from strictured CD than in those from non-strictured CD (*P*=0.0005) and normal gut (*P*=0.001).

### IL-17RC and IL-17RB expression on myofibroblasts

**Figure 4:**
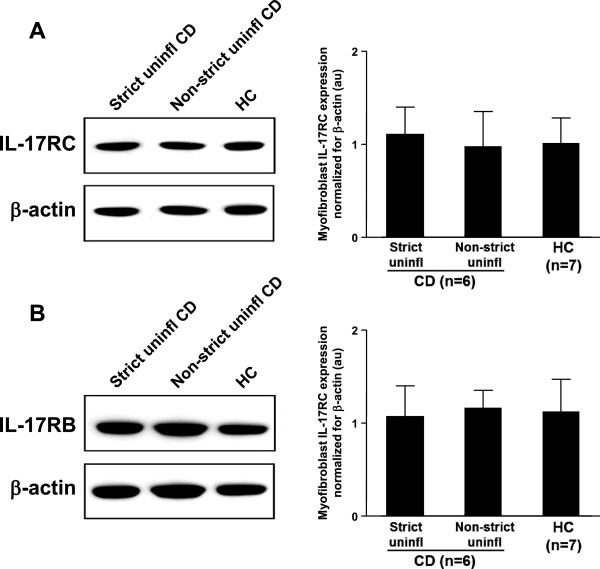
**Expression of interleukin (IL)-17A and IL-17E receptors on intestinal myofibroblasts. (A)** IL-17RC and **(B)** IL-17RB were detected by immunoblotting on lysates of myofibroblasts isolated from uninflamed areas of strictured (Strict uninfl) and non-strictured (Non-strict uninfl) gut of six patients with fibrostenosing Crohn’s disease (CD) and from normal gut of seven healthy control (HC) subjects. Each example shown in the left panels is representative of experiments performed in all patients with CD and all HC subjects. Blots were stripped and analyzed for β-actin as an internal loading control. In the right panels, densitometry of IL-17RC and IL-17RB expression normalized for β-actin is shown. Results are mean ± SEM. au, Arbitrary units.

We then analyzed IL-17RC and IL-17RB expression by immunoblotting in lysates of myofibroblasts isolated from uninflamed areas of strictured and non-strictured gut of six patients with fibrostenosing CD, and from normal gut of seven control subjects. Myofibroblasts isolated from strictured CD areas, non-strictured CD areas, and control gut expressed both IL-17RC (Figure 4A) and IL-17RB (Figure 4B). However, no significant difference in IL-17RC and IL-17RB expression was found between strictured, non-strictured CD, and control myofibroblasts.

### *In vitro* effect of IL-17A and IL-17E on matrix metalloproteinase (MMP)-3, MMP-12, and TIMP-1 production by myofibroblasts

**Figure 5:**
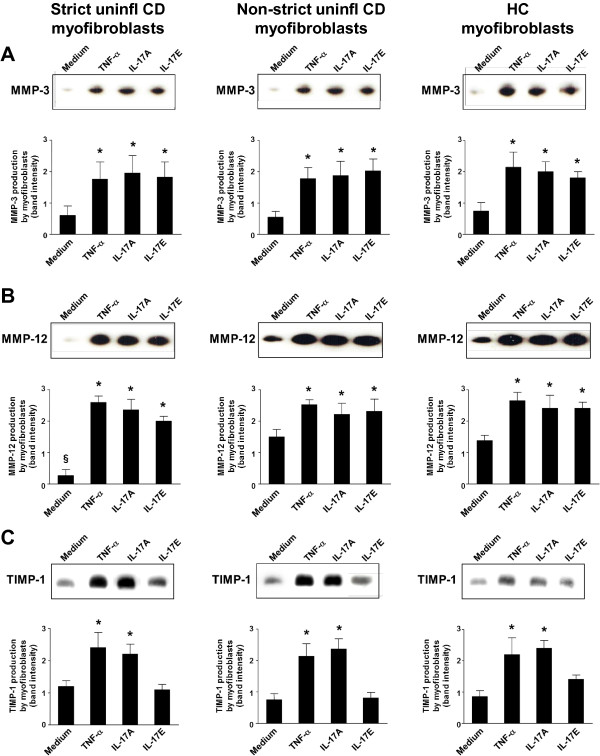
**Effect of interleukin (IL)-17A and IL-17E on the production of matrix metalloproteinase (MMP)-3, MMP-12, and tissue inhibitor of metalloproteinase (TIMP)-1 by intestinal myofibroblasts. (A)** MMP-3, **(B)** MMP-12, and **(C)** (TIMP-1 in culture supernatants of myofibroblasts isolated from uninflamed areas of strictured (Strict uninfl) and non-strictured (Non-strict uninfl) gut of six patients with fibrostenosing Crohn’s disease (CD) and from normal gut of six healthy control (HC) subjects, cultured for 24 hours with medium alone or recombinant human (rh) tumor necrosis factor (TNF)-α, or rhIL-17A, or rhIL-17E. Blots are representative of experiments performed in all patients with CD and HC subjects. Lower panels show densitometry of western blots. Values are mean ± SEM. *P<0.05 versus myofibroblasts from the same study group cultured with medium alone. ^§^*P*<0.05 versus Non-strict uninfl CD and HC myofibroblasts cultured under the same conditions.

Matrix metalloproteinase (MMP)-3, MMP-12, and TIMP-1 production was evaluated by immunoblotting in culture supernatants of myofibroblasts isolated from uninflamed areas of strictured or non-strictured gut of six patients with fibrostenosing CD, and from normal gut of six control subjects, and subsequently stimulated *in vitro* with recombinant human (rh)TNF-α, rhIL-17A, or rhIL-17E. rhIL-17A and rhIL-17E induced a significant (*P*<0.05) increase in both MMP-3 and MMP-12 production by myofibroblasts from strictured and non-strictured areas of patients with CD and from normal gut of control subjects (Figure 5A,B). rhTNF-α induced a significant (*P*<0.05) increase in both MMP-3 and MMP-12 production compared with control (myofibroblasts from the same group of patients cultured with medium alone). No significant difference in MMP-3 or MMP-12 production was found between rhTNF-α, rhIL-17A, and rhIL-17E stimulation in the three conditions studied. Myofibroblasts from CD strictured areas showed a significantly (*P*<0.05) lower spontaneous release of MMP-12 than myofibroblasts from CD non-strictured areas or control gut. Myofibroblast stimulation with rhIL-17A induced a significant (*P*<0.05) increase in TIMP-1 production compared with unstimulated cells from the same group of patients (Figure 5C). rhIL-17E did not induce any significant change in TIMP-1 production compared with myofibroblasts from the same group of patients cultured with medium alone. rhTNF-α induced a significant (*P*<0.05) increase in TIMP-1 production compared with myofibroblasts from the same group of patients cultured with medium alone. No significant difference in TIMP-1 production was found between rhTNF-α and rhIL-17A stimulation in all the three conditions studied.

### *In vitro* effect of IL-17A and IL-17E on myofibroblast collagen production

**Figure 6:**
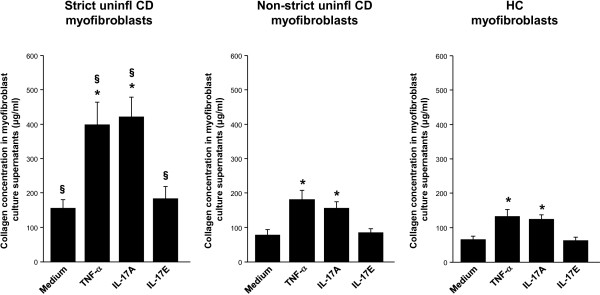
**Effect of interleukin (IL)-17A and IL-17E on the production of collagen by intestinal myofibroblasts.** Levels of collagen, expressed as μg/ml, in the supernatants of myofibroblasts isolated from uninflamed areas of strictured (Strict uninfl) and non-strictured (Non-strict uninfl) gut of six patients with fibrostenosing Crohn’s disease (CD) and from normal gut of six healthy control (HC) subjects, cultured for 24 hours with medium alone or recombinant human (rh)tumor necrosis factor (TNF)-α, or rhIL-17A, or rhIL-17E. Values are mean ± SEM. *P<0.05 versus myofibroblasts from the same study group cultured with medium alone. ^§^*P*<0.05 versus Non-strict uninfl CD and HC myofibroblasts cultured under the same conditions.

We cultured myofibroblasts isolated from uninflamed areas of strictured and non-strictured gut of six patients with fibrostenosing CD, and from normal gut of six control subjects, in the presence or absence of rhTNF-α, rhIL-17A, or rhIL-17E, and measured collagen in the culture supernatants (Figure 6). Myofibroblast stimulation with rhIL-17A induced a significant (*P*<0.05) increase in mean collagen production (strictured CD myofibroblasts: 412 ± 57 μg/ml; non-strictured CD myofibroblasts: 154 ± 17 μg/ml; control myofibroblasts: 114 ± 12 μg/ml) compared with cells from the same group of patients cultured with medium alone (strictured CD myofibroblasts: 155 ± 23 μg/ml; non-strictured CD myofibroblasts: 76 ± 15 μg/ml; control myofibroblasts: 67 ± 7 μg/ml). rhIL-17E did not induce any significant change in mean collagen production by strictured CD myofibroblasts (181 ± 37 μg/ml), non-strictured CD myofibroblasts (83 ± 7 μg/ml) or control myofibroblasts (61 ± 10 μg/ml) compared with unstimulated cells from the same group of patients. rhTNF-α induced a significant (*P*<0.05) increase in mean collagen production by strictured CD myofibroblasts (399 ± 62 μg/ml), non-strictured CD myofibroblasts (175 ± 22 μg/ml) and control myofibroblasts (124 ± 18 μg/ml) compared with unstimulated cells from the same group of patients. No significant difference in collagen production was found between cells cultured with rhTNF-α and rhIL-17A. Strictured CD myofibroblasts produced significantly (*P*<0.05) higher amounts of collagen than did non-strictured CD and control myofibroblasts cultured under the same conditions. No significant difference in collagen production was found between non-strictured CD and control myofibroblasts cultured under the same conditions.

### *In vitro* effect of IL-17A and IL-17E on myofibroblast migration

**Figure 7:**
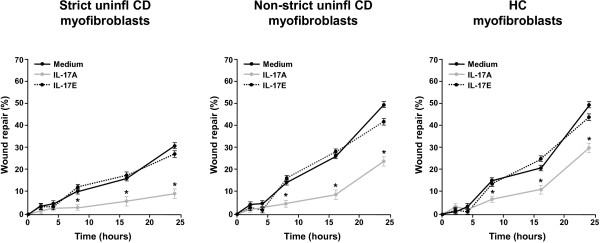
**Wound-healing scratch assay.** Effect of recombinant human (rh)IL-17A and rhIL-17E on the migration, assessed by an *in vitro* wound-healing scratch assay, of myofibroblasts isolated from uninflamed areas of strictured (Strict uninfl) and non-strictured (Non-strict uninfl) gut of six patients with fibrostenosing Crohn’s disease (CD) and from normal gut of seven healthy control (HC) subjects. Myofibroblasts were cultured with rhIL-17A or rhIL-17E or medium alone. Results, expressed as percentage of wound repair, are mean ± SEM. *P<0.05 versus myofibroblasts cultured with medium only at 8, 16, and 24 hours.

To evaluate the role of IL-17A and IL-17E on myofibroblast migration, a wound-healing scratch assay was performed using subconfluent monolayers of myofibroblasts isolated from uninflamed areas of strictured and non-strictured gut of six patients with fibrostenosing CD, and from normal gut of seven control subjects. Cell migration was measured as the percentage of wound repair and results were expressed as mean percentage of wound repair (see Methods section). rhIL-17A significantly (*P*<0.05) reduced migration of strictured CD, non-strictured CD and control myofibroblasts at 8 hours (3.2 ± 1.1%, 4.6 ± 1.0%, and 6.4 ± 1.6%, respectively), 16 hours (5.8 ± 1.7%, 8.4 ± 1.8%, and 10.6 ± 1.9%, respectively) and 24 hours (8.8 ± 1.9%, 23.2 ± 2.3%, and 29.7 ± 2.0%, respectively) compared with myofibroblasts from the same three groups cultured with medium alone and evaluated at the same time points (8 hours 9.8 ± 1.3%, 13.7 ± 1.8%, and 14.6 ± 1.7%, respectively; 16 hours: 15.6 ± 1.6%, 25.3 ± 2.3%, and 20.3 ± 1.1%, respectively; and 24 hours: 30.6 ± 2.3%, 49.1 ± 1.9%,and 48.4 ± 1.8%, respectively) (Figure 7). Compared with medium alone, rhIL-17E did not induce any significant change in strictured CD, non-strictured CD, and control myofibroblast migration (8 hours: 11.2 ± 1.2%, 15.4 ± 1.4%, 12.9 ± 1.5%, respectively; 16 hours: 16.2 ± 1.4%, 28.2 ± 1.7%, 25.1 ± 1.1%, respectively; and 24 hours: 26.3 ± 1.9%, 41.3 ± 2.2%, 43.4 ± 2.2%, respectively).

## Discussion

In the present study, we found that IL-17A was overexpressed in CD strictures compared with non-strictured CD areas and control gut. We also found that myofibroblasts from CD strictures, which express the IL-17A receptor(IL-17RC), responded to IL-17A by producing more collagen and TIMP-1, and had reduced migratory ability. By contrast, IL-17E was not upregulated in CD strictures, and it did not influence collagen and TIMP-1 production or migration of myofibroblasts from CD strictures.

IL-17A, which is produced mainly by Th1 and Th1/Th17 cells [18,19], mediates autoimmunity and immune defense against pathogens [20], and is increased in the intestinal mucosa of patients affected by chronic inflammatory bowel disorders, such as celiac disease [21], CD, and ulcerative colitis [22,23]. Moreover, experimental studies have shown an important role for IL-17A in tissue remodeling and fibrosis in a number of different tissues. In particular, IL-1β-induced and bleomycin-induced lung fibrosis seems to depend on the action of IL-17A, and in addition, IL-17A^−/−^ mice are less susceptible to experimental skin fibrosis [7,24]. As opposed to acute experimental colitis, which is mainly characterized by a Th1 immune response, chronic trinitrobenzenesulfonic acid (TNBS)-induced murine colitis, which is accompanied by intestinal fibrosis, is driven predominantly by IL-17A-producing Th17 cells [25]. The observation that targeting IL-23 (a key cytokine in Th17 cell development) with a p40 peptide-based vaccine ameliorates chronic TNBS-induced colitis,and reduces IL-17A, TGF-β1 levels, and collagen deposition in the bowel wall [25], highlights the importance of IL-17A in intestinal experimental fibrosis.

In the current study, we found, using immunoblotting and ELISA, that IL-17A was overexpressed *in vivo* in strictured CD gut compared with non-strictured CD and control gut. We decided to collect samples from uninflamed areas of patients with fibrostenosing CD so that any confounding effect of inflammation on IL-17A was minimized. Similar to the *in vivo* data, *ex vivo* tissue explants from CD strictures produced more IL-17A than explants from non-strictured CD areas and control gut. In keeping with our previous results [16], collagen content and TGF-β1 transcripts in tissue explants from uninflamed CD strictures were also increased compared with uninflamed non-strictured CD areas and control gut. The recent observation that IL-17A expression is higher in long-standing CD mucosa compared with early mucosal lesions [26] further strengthens our findings, because intestinal fibrosis is a late-stage process in CD.

On the basis of our findings showing that CD myofibroblasts expressed the IL-17A receptor IL-17RC we decided to stimulate these cells with IL-17A in *in vitro* experiments. In keeping with the study of Bamba *et a*l. [27], we found that IL-17A increased MMP-3 and MMP-12 production by myofibroblasts from CD strictured and non-strictured areas. However, in parallel, IL-17A upregulated TIMP-1 and collagen release, and reduced the migration ability of CD myofibroblasts. In a murine model of intestinal fibrosis, TIMP-1, which is upregulated, effectively inhibited ECM degradation by MMPs [28], and TIMP-1 was found to be increased in collagenous colitis and in CD strictures [16,29]. Taken together, our data support a pro-fibrogenic role for IL-17A in CD intestinal fibrosis.

In parallel to studying the role of IL-17A, we also investigated the influence of IL-17E in the fibrogenic process in CD. IL-17E exerts two distinct immunological functions: on the one hand, it promotes Th2 response in allergic diseases including asthma [30], while on the other hand, it dampens the inflammatory process in immune-mediated disorders including IBD [31]. A pro-fibrogenic role for IL-17E in experimental fibrotic disorders has also been shown. In particular, IL-17E mediates pulmonary collagen deposition in mice exposed to house dust mite [10], and intestinal TNBS-induced fibrosis was found to be associated in the early phase by a marked increase in IL-17E [32]. When we investigated IL-17E *in vivo*, we found no difference in the expression of IL-17E in uninflamed CD strictures, uninflamed non-strictured CD areas, and control gut. Likewise, *ex vivo* tissue explants from the same three groups of patients produced comparable amounts of IL-17E. The IL-17E receptor IL-17RB was expressed at a similar level in strictured, non-strictured CD, and control gut. IL-17RB was found to be expressed by CD myofibroblasts, and stimulation with IL-17E increased MMP-3 and MMP-12 production by myofibroblasts from CD strictured and non-strictured areas and from control subjects. However, we did not observe any influence of IL-17E on collagen or TIMP-1 production by CD myofibroblasts or on their migration ability.

## Conclusions

In conclusion, our findings provide evidence that IL-17A, but not IL-17E, may have a pro-fibrogenic role in CD. Clinical studies are needed to ascertain whether therapeutic blockade of IL-17A through the monoclonal anti-IL-17A antibody secukinumab [33] might offer a chance to counteract the fibrogenic process in patients with CD. However, we must emphasize that the recent findings that anti-IL-17A exacerbates CD [33] will mean that patient selection for such treatment will be crucial.

## Methods

### Ethics approval

This study was approved by the local Ethics Committee (NRES Committee London - City & East). Each patient who took part in the study gave informed consent to participate in the study and to publish the results deriving from this research. All the experiments reported in this study are in compliance with the Helsinki Declaration.

### Patients and tissues

Surgical specimens were taken from uninflamed areas of strictured and non-strictured ileum or colon of 29 patients with fibrostenosing CD (Table 1). Because the excessive collagen deposition occurs in the sub-mucosa and outer muscle layers, we discarded the mucosa upon dissection, and used the muscle-enriched fraction. Diagnosis of CD was ascertained by standard clinical criteria [34], and strictured areas were identified by enteroclysis [35]. None of the patients with CD had been treated previously with ciclosporin, tacrolimus, methotrexate, or anti-TNF-α antibodies. Intestinal samples were also collected from macroscopically and microscopically unaffected (at least 500 mm from the tumoral mass) ileum or colon of 27 patients undergoing intestinal resection for colon cancer (mean age 57 years, range 45–71 years). The intestinal tissue was homogenized and used in immunoblotting experiments, or processed to isolate myofibroblasts, or cultured *ex vivo*.

**Table 1:** Clinical features of patients with fibrostenosing Crohn’s disease (n=29)

**Characteristics and parameters**	**n**	**Median (range)**
Age, years		35.8 (23 to 61)
First attack	6	
Intestinal location		
Small bowel and colon	15	
Small bowel only	9	
Colon only	5	
Duration of disease, months		81.2 (7 to 196)
Number of recurrences		4 (0 to 9)
CDAI		223 (155 to 426)
Treatment		
Mesalazine	13	
Topical steroids	3	
Antibiotics	9	
Azathioprine/6-MP/methotrexate	14	

Abbreviations: *CDAI* Crohn’s Disease Activity Index, *6-MP* 6-mercaptopurine.

### Organ culture

Intestinal tissue explants (1 mm^3^ in size) were placed in 12-well tissue culture plates (BD Biosciences, Oxford, UK; one explant per well) and cultured at 37°C and 5%CO_2_ in 800 μl serum-free HL-1 medium (Cambrex Bio Science, Wokingham, UK) supplemented with 100 U/ml penicillin and 100 μg/ml streptomycin, as previously described [36]. After 24 hours *ex vivo* culture, supernatants were collected and stored at −70°C until used for cytokine measurement by ELISA, and intestinal explants were homogenized for subsequent evaluation of TGF-β1 expression by quantitative reverse transcription (qRT)-PCR.

### Myofibroblast isolation and culture

The mucosa was dissected away from full thickness samples of bowel and discarded. The remaining tissue was cut into 1 mm-sized cubes and cultured at 37°C in a humidified CO_2_ incubator in Dulbecco’s modified Eagle’s medium (DMEM; Sigma-Aldrich, Poole, UK) supplemented with 20% fetal bovine serum (FBS), 1% non-essential amino acids (Invitrogen Ltd., Paisley, UK), 100 U/ml penicillin, 100 μg/ml streptomycin, 50 μg/ml gentamycin, and 1 μg/ml amphotericin (Sigma-Aldrich). Myofibroblasts migrated out of the tissue after a few days. Established colonies of myofibroblasts were seeded into 25-cm^2^ culture flasks and cultured in DMEM supplemented with 20% FBS and antibiotics. At confluence, the cells were passaged using trypsin-EDTA in a 1:2 to 1:3 split ratio. Cells were grown to at least passage 4 before there were enough to use in stimulation experiments, and were characterized by immunocytochemical staining, as previously described [37]. Subconfluent monolayers of myofibroblasts seeded in 12-well plates at 3×10^5^ cells per well were starved in serum-free medium for 24 hours at 37°C and 5%CO_2_ before culture for 24 hours at 37°C and 5%CO_2_ with serum-free DMEM containing antibiotics in the absence or presence of 10 ng/ml of rhIL-17A, or rhIL-17E, or rhTNF-α (all R&D Systems, Abingdon, Oxfordshire, UK).

### Wound-healing scratch assay

Myofibroblast migration was assessed in accordance with the modified method of Rodriguez *et al*. [16,38]. Briefly, cells (2×10^5^) were seeded into cell culture dishes (Nunc; Nalge Nunc International, Rochester, NY, USA) with 2 mm grids, size 35×10 mm, in 2 ml of DMEM supplemented with 20% FBS and antibiotics. The cells were maintained at 37°C and 5% CO_2_ until confluent. Once confluent, each dish of monolayer cells was given a mechanical wound by scoring with a 200 μl pipette tip, parallel to the grid bars along the central grid line. This permitted easy viewing of the cells growing back together, and ensured that the 2 mm grid could be used as a reference, so that the wound areas could be measured and compared. Wound placement was checked with an inverted microscope (CK2; Olympus UK Ltd, London, UK). The medium was then removed, and the cells were washed five times with HL-1 serum-free medium (Cambrex Bio Science) supplemented with antibiotics, and then replaced with 1.5 ml HL-1 medium with or without 10 ng/ml rhIL-17A or rhIL-17E (both R&D Systems). Photographs of the cells in each grid along the induced wound were taken at 0, 2, 4, 8, 16 and 24 hours, using a digital camera (Camedia; Olympus UK Ltd) with 34 to 40 zoom, and 20× magnification, attached to a light microscope. The computer program Image J was used to measure the area of initial damage (images taken at time 0) and of the remaining damage at subsequent time points. Each grid image was observed separately, and two points per grid at the same position at every time point were measured using imaging software at the same magnification. The percentage of wound repair was then calculated.

### ELISA

Concentrations of IL-17A and IL-17E in tissue sample homogenates were measured using specific ELISA kits (R&D Systems), in accordance with the manufacturer’s instructions, and were normalized to the total protein concentration, determined by a protein assay (Bio-Rad Laboratories, Hemel Hempstead, UK). Concentrations of IL-17A, IL-17E, IL-6, and TNF-α in organ-culture supernatants were assessed using specific ELISA kits (R&D Systems), in accordance with the manufacturer’s instructions.

### RNA extraction and analysis of mRNA expression by qRT-PCR

RNA was extracted from cultured tissue explants. cDNAs were synthesized (Improm-II RT system; Promega, Southampton, Hampshire, UK) using random hexamers and 1 μg of RNA in a final volume of 20 μl. RT reactions were performed using the Improm-II reverse transcriptase enzyme from the kit. An RT reaction without reverse transcriptase enzyme was performed for each tissue type as a negative control for qPCR. TGF-β1 primers and probe sets were validated for use with the ^∆∆^C_t_ method of quantification. The probe was labeled with a 59-reporter dye FAM (6-carboxy-fluorescein) and the 39-quencher dye TAMRA (6-carboxy-N,N,N’,N9-tetramethyl-rhodamine). RT reactions were diluted 1 in 10 in distilled H_2_O, and 5 μl of template was added to 6.5 μl of 2× master mix (Eurogentech, Seraing, Belgium) containing 1.2 μmol/l forward and reverse primers and 0.248 μmol/l of probe in a total volume of 12.5 μl. The PCR protocol was as follows: 50°C for 2 minutes and 95°C for 10 minutes, followed by 40 cycles of denaturation at 95°C for 15 seconds, and annealing/extension at 60°C for 1 minute. Thermocycling and real-time detection of PCR products were performed on asequence detection system (iCycler iQ; Bio-Rad Laboratories). Expression levels were normalized against 18S, β-actin, and glyceraldehyde 3-phosphate dehydrogenase (GAPDH), and values were calculated using the ∆∆C_t_ method.

### Collagen assay

Total soluble forms of collagen were measured in supernatants of tissue explants and myofibroblasts (Sircol Collagen Assay Kit; Biocolor Ltd, Belfast, UK), in accordance with the manufacturers’ instructions. The collagen content in each sample was calculated as an average of three readings.

### Western blotting

Western blotting was performed using a modification of a previously described method [36]. Tissue samples or myofibroblasts were homogenized in ice-cold lysis buffer, and the amount of protein was determined by a protein assay (Bio-Rad Laboratories). Equal amounts of protein or 15 μl of cell- culture supernatants were loaded into each lane and run in 10% sodium dodecyl sulphate-polyacrylamide gel electrophoresis (SDS-PAGE) gels under reducing conditions. After electrophoresis, protein was transferred onto nitrocellulose membranes (Bio-Rad Laboratories). Membranes were blocked with 5% non-fat dry milk, followed by incubation overnight at 4°C with the following antibodies: goat anti-human IL-17A (1 μg/ml), goat anti-human IL-17E (0.1 μg/ml) (both R&D Systems), mouse anti-human IL-17RB (1:500 dilution; LifeSpan Biosciences, Seattle, WA), mouse anti-human IL-17RC (1:750 dilution), rabbit anti-human MMP-3 (1:200 dilution), rabbit anti-human MMP-12 (1:1000 dilution) (all three from Abcam Ltd, Cambridge, UK), or mouse anti-human TIMP-1 (1 μg/ml; Oncogene Research, Nottingham, UK). Appropriate antibodies conjugated to horseradish peroxidase (Dako, High Wycombe, Buckinghamshire, UK) were used as secondary antibodies, and the reaction was developed with enhanced chemiluminescence (ECL Plus Kit;Amersham Biosciences, Little Chalfont, Buckinghamshire, UK). Blots were then stripped and analyzed for β-actin, as an internal loading control, using a rabbit anti-β-actin antibody (1:5000 dilution; Abcam Ltd). Bands were quantified by scanning densitometry (LKB Ultrascan XL Laser Densitometer; Kodak, Hemel Hempstead, UK).

### Statistical analysis

Data were analyzed by the GraphPad Prism statistical PC program (GraphPad Software, San Diego, CA) using the paired *t* test and the Mann–Whitney *U*-test. *P*<0.05 was considered significant.

## Abbreviations

CD: Crohn’s disease; DMEM: Dulbecco’s modified Eagle’s medium; ECM: Extracellular matrix; FBS: Fetal bovine serum; GAPDH: Glyceraldehyde 3-phosphate dehydrogenase; IL: Interleukin; IBD: Inflammatory bowel disease; MMP: Matrix metalloproteinase; qRT: Quantitative reverse transcription; rh: Recombinant human; SDS-PAGE: Sodium dodecyl sulphate-polyacrylamide gel electrophoresis; TGF: Transforming growth factor; Th: T helper; TIMP: Tissue inhibitor of metalloproteinase; TNBS: Trinitrobenzenesulfonic acid; TNF: Tumor necrosis factor.

## Competing interests

The authors declare that they have no competing interests.

## Authors’ contributions

ADS, PB, and TTM designed the study; PB, GS, SA, AG, and ADS were involved in the recruitment and collection of samples; PB, SLFP, FA, PG, RC, and AP performed all laboratory work; PB, PG, and ADS performed data analysis. Writing of the manuscript was carried out by PB and ADS. GM, SLFP, GRC, and TTM provided scientific advice throughout the study, and edited the manuscript prior to submission. All authors read and approved the final manuscript.
